# On the Contagion of Puerperal Fever

**Published:** 1855-06

**Authors:** J. Cheston Morris

**Affiliations:** Philadelphia


### *On the Contagion of Puerperal Fever.*

To the Editor of the Medical Examiner:

*Dear Sir,*—On reading the review, in the March number of the Examiner, of Dr. Holmes' work, I was impressed with the conviction that it must be decided upon experience alone, whether puerperal fever is contagious or not, and whether there are not different diseases included under that name. This experience should be generally contributed by all who have cases in their charge, and then the professional mind would soon become settled on this now much mooted question. Thinking that perhaps a statement of four obstetrical cases that have been under my care lately might prove useful, I subjoin it.

Mrs. McT. was brought to bed with her first child, Dec. 4th, 1854. After a natural labor she was delivered of a son; considerable hemorrhage followed his birth, which was easily arrested however. On the next day she was seized with a chill, followed by fever, with pulse of 126; slight chills recurred for two days more; retention of urine took place also. On the fourth day tenderness was observable in the hypogastric region and slight meteorism; there was little or no milk in the breasts, and the pulse rose to 144. The tenderness increased for some days, and the abdomen became tympanitic; palpitations became troublesome; but no delirium or heavy perspirations were observable. By the steady use of blue mass and opium (not however to narcotism or ptyalism) until the skin began to relax, and the tongue, previously heavily furred, showed a disposition to clean, and supporting the system then with quinine, she improved much, and was beginning to walk about the room by December 22, but on December 25 was seized with phlegmasia alba in the right leg. Her pulse rose again to 140, she became delirious, and had copious sweats, but under the influence of quinine she recovered rapidly, and by the 20th of January, 1855, was able to leave her room.

On January 11th I was sent for to attend Mrs. T., living two squares only from Mrs. McT. She was safely delivered of a boy and got well without a bad symptom. She had had puerperal fever after her previous child's birth.

My next obstetric case occurred on March 9th. Mrs. F., of nervous temperament, was delivered of a healthy girl after a short labor. She had for some time been suffering under a white discharge, which I suspected was syphilitic, but owing to absence from the city was unable to make a specular examination. On the second day this patient had a slight chill, which returned with increased severity on the third and fourth days. Intense peritonitis now set in, with a pulse of 140, a heavily furred tongue becoming dry and dark in the centre, changed expression of countenance, slight but well marked intoxicated delirium, and heavy sweats. Slight retention of urine also took place. But under the use of blue mass and opium at first, then of oil of turpentine (as a stimulant) and laudanum and quinine, she gradually recovered, and is now suffering only from weakness.

From the bedside of this patient I was called to see Mrs. M., living two miles from the last patient, on March 16th. I drew off Mrs. F.'s urine with the catheter, and then drove immediately to Mrs. M.'s and delivered her of a fine girl. The mother was well in a few days.

Such is the history of my obstetric cases between December and March. Previous to December, nothing of interest had occurred in them, all getting well speedily. The alternation of the cases, and the almost direct communication established between the last two, seem to me worthy of attention.

Yours respectfully,

J. Cheston Morris, M.D.

*Philadelphia, April 20th,* 1853.

